# Switch-on Fluorescence Analysis of Protease Activity with the Assistance of a Nickel Ion-Nitrilotriacetic Acid-Conjugated Magnetic Nanoparticle

**DOI:** 10.3390/molecules28083426

**Published:** 2023-04-13

**Authors:** Xiaohua Ma, Yingxin Lv, Panpan Liu, Yuanqiang Hao, Ning Xia

**Affiliations:** 1 Henan Key Laboratory of Biomolecular Recognition and Sensing, Shangqiu Normal University, Shangqiu 476000, China; 2 College of Chemistry and Chemical Engineering, Anyang Normal University, Anyang 455000, China

**Keywords:** proteases, caspase-3, nitrilotriacetic acid, homogeneous assays, fluorescence, magnetic nanoparticle

## Abstract

Heterogeneous protease biosensors show high sensitivity and selectivity but usually require the immobilization of peptide substrates on a solid interface. Such methods exhibit the disadvantages of complex immobilization steps and low enzymatic efficiency induced by steric hindrance. In this work, we proposed an immobilization-free strategy for protease detection with high simplicity, sensitivity and selectivity. Specifically, a single-labeled peptide with oligohistidine-tag (His-tag) was designed as the protease substrate, which can be captured by a nickel ion-nitrilotriacetic acid (Ni-NTA)-conjugated magnetic nanoparticle (MNP) through the coordination interaction between His-tag and Ni-NTA. When the peptide was digested by protease in a homogeneous solution, the signal-labeled segment was released from the substrate. The unreacted peptide substrates could be removed by Ni-NTA-MNP, and the released segments remained in solution to emit strong fluorescence. The method was used to determine protease of caspase-3 with a low detection limit (4 pg/mL). By changing the peptide sequence and signal reporters, the proposal could be used to develop novel homogeneous biosensors for the detection of other proteases.

## 1. Introduction

Proteases participate in many important physiological processes, such as protein digestion, blood coagulation, and immune system activation. The occurrence of some diseases is closely related to the abnormal activity of proteases [1,2,3,4,5]. For example, matrix metalloproteinases are overexpressed in some cancers, serine proteases are involved in the closely coordinated coagulation cascade, human immunodeficiency virus (HIV) proteases are overexpressed in the life cycle of HIV patients, SARS-CoV-2 main proteases are related to COVID-19, and β/γ-secretases are responsible for the production of toxic β-amyloid peptides in the brains of patients with Alzheimer’s disease. Some diseases can be treated well by regulating the activity of proteases with inhibitor drugs. Therefore, the development of simple, sensitive, low cost, and high-throughput methods for monitoring protease activity is of great significance for early diagnosis and effective treatment of protease-related diseases.

Proteases can catalyze the hydrolysis of proteins or peptides and decompose them into amino acids or small peptide fragments. The methods for analysis of protease activity usually include homogenous and heterogeneous assays, such as liquid chromatography, mass spectrometry, electrochemistry, colorimetry, fluorescence, surface plasmon resonance and so on [4,6,7,8,9,10,11,12,13,14,15,16,17]. Among these, fluorescence-based homogenous analysis is the most commonly used method for protease detection because of its high sensitivity and quantitative results. In this method, the substrate peptide is often modified with a pair of quencher and fluorophore groups [18,19,20,21,22,23]. The hydrolysis of peptides can lead to the separation of quenchers and fluorophores, leading to the increase in the fluorescence signal. Although this fluorescence method has the characteristics of high sensitivity and selectivity, dual-labeling of peptides at both terminals may increase the cost and complexity of substrate synthesis, limit the protease to approach the cleavage site and reduce the cleavage efficiency of proteolysis [24,25,26,27,28]. Recently, single-labeled fluorescent nucleotide probes have been widely used for the detection of metal ions and nucleic acids with the advantages of low cost and high sensitivity [29,30]. However, there are few reports on the general detection of protease activity using single-labeled peptide substrates [31].

Nanomaterials such as gold nanoparticles, carbon nanotubes and graphene oxide as quenchers have been integrated with single-labeled fluorescent peptides for monitoring protease activity through the fluorescence resonance energy transfer (FRET) effect [32,33,34,35,36,37]. The nanomaterials usually exhibit superior quenching ability to molecular quenchers due to the long-range energy transfer. However, immobilization of peptide substrates on the solid surface may cause the steric hindrance effect to affect the configurational freedom of peptides, thus limiting their interaction with proteases and decreasing the cleavage efficiency [38,39]. In addition, peptide immobilization requires laborious and time-consuming procedures. In this respect, it is of paramount importance to the manipulation of external surfaces for anchoring peptide substrates. Oligohistidine-tag (His-tag) can be easily integrated into peptides or proteins through synthetic or recombinant techniques. The nickel ion-nitrilotriacetic acid (Ni-NTA) complex shows high-affinity toward His-tag in which four coordination sites are occupied by NTA and two of them are coordinated with two imidazole moieties of His-tag [40,41,42]. Based on strong and specific interactions, Ni-NTA-conjugated polymers, magnetic beads and silica nanoparticles have been widely applied for the separation, purification and delivery of peptides and proteins with His-tags [41,43,44,45,46]. In this work, the commercialized Ni-NTA-conjugated magnetic nanoparticles (MNPs) were employed for the development of a switch-on fluorescent biosensor for monitoring protease activity with Caspase-3 (Cas3) as an example. Fluorescently-labeled peptide substrates with His-tags can be captured by Ni-NTA-MNPs through the interactions between His-tag and Ni-NTA, leading to adecrease in fluorescence. When peptide substrates were degraded using the proteolysis, the fluorophore-included peptide segments were released from the peptide substrates or the peptide-conjugated NTA-Ni-MNPs, thus turning on the fluorescence of solution. The reaction rate of proteolysis for the peptides dispersed in solution and immobilized on the surface of NTA-Ni-MNPs was investigated. This strategy can be used for the detection of various proteases by matching the peptide sequences specific to the targets. In addition, by replacing the fluorophores with other signal molecules such as electroactive molecules, enzymes and nanoparticles, single-labeled peptides could be used for electrochemical or colorimetric analysis of the activity of various proteases.

## 2. Results and Discussion

### 2.1. Principle of This Proposal

**Scheme 1:**
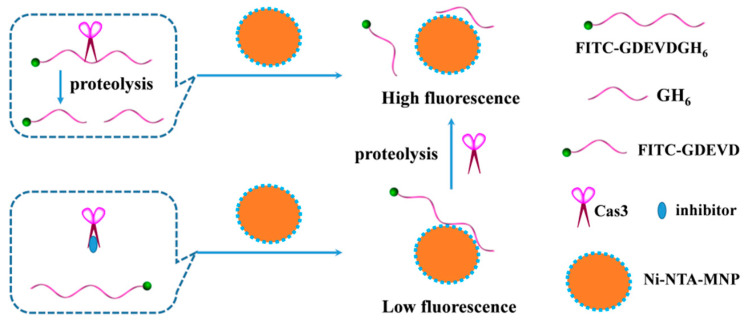
Schematic illustration of the method for Cas3 detection.

Cas3, an important cysteine protease of the caspase family, plays a critical role in the cell apoptosis pathway [47,48]. To verify the analytical performances of this proposal, Cas3 was used as the model analyte. The principle of magnetically assisted assays of Cas3 is shown in Scheme 1. His-tag (HHHHHH or H_6_) was included in the C-terminal of a Cas3-recognized peptide sequence (DEVD), and fluorescein isothiocyanate (FITC) was labeled in the N-terminal of the peptide. The peptide substrate of FITC-GDEVDGH_6_ could be captured and removed by Ni-NTA-MNP through the interaction between His-tag and Ni-NTA, thus quenching the fluorescence. Once the peptide was selectively digested by Cas3 in solution, the FITC-labeled segment (FITC-GDEVD) was released from the substrate. The released segment could not be removed by Ni-NTA-MNP even under the magnetic force and thus emitted a strong fluorescence. Inhibiting the activity of Cas3 with a potential inhibitor could prevent the cleavage of the substrate peptide and thus cause the decrease in the fluorescence signal. The fluorescence intensity was proportional to the concentration and activity of Cas3, allowing for the detection of Cas3 and screening of its inhibitor drugs. The strategy is simple and sensitive because it does not require the pre-immobilization of the peptide substrate on the solid surface, thus eliminating the steric hindrance effect and improving the cleavage efficiency.

### 2.2. Feasibility for Cas3 Detection

**Figure 1:**
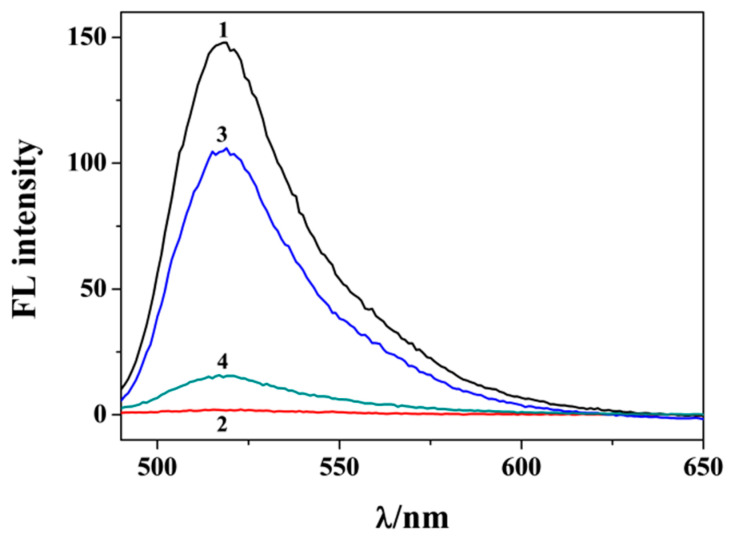
Fluorescence spectra of different systems: FITC-GDEVDGH_6_ (curve 1), FITC-GDEVDGH_6_ + Ni-NTA-MNP (curve 2) and FITC-GDEVDGH_6_/Cas3 + Ni-NTA-MNP (curve 3). Curve 4 corresponds to that of incubation of FITC-GDEVDGH_6_/Ni-NTA-MNP with Cas3. The concentrations of FITC-GDEVDGH_6_ and Cas3 used were 500 nM and 100 ng/mL.

Figure 1 shows the results to confirm the validity of the proposal. The fluorescence intensity of the peptide substrate decreased significantly after being incubated with Ni-NTA-MNP under a magnetic force (c.f. curves 1 and 2). The change was time-independent, indicating a rapid coordination interaction between His_6_ tag and Ni-NTA. However, no obvious change in the fluorescence signal was observed when the H_6_-free peptide (FITC-GDEVD) was incubated with Ni-NTA-MNP at the same condition. The results indicated that the peptide substrate was captured and removed by Ni-NTA-MNP through the interaction between His-tag and Ni-NTA. Interestingly, when the peptide substrate was incubated with Cas3 and then treated with Ni-NTA-MNP, the fluorescence signal increased greatly (curve 3). The result confirmed that the peptide probe could be enzymatically cleaved with Cas3, thus leaving the FITC-GDEVD segment in solution for determination. To evaluate the influence of steric hindrance on the cleavage efficiency, the FITC-GDEVDGH_6_ substrates were immobilized on the Ni-NTA-MNP surface and then digested by Cas3 at the same experimental conditions. Consequently, the fluorescence intensity (curve 4) was lower than that achieved by the immobilization-free method (curve 3), suggesting that the immobilization of the peptide on the solid surface decreased the cleavage efficiency to some extent. The problem may be resolved by adding a spacer between the solid surface and the cleavage core of the peptide or by employing small-size nanoparticles to load the peptide substrate.

### 2.3. Optimization of Experimental Conditions

**Figure 2:**
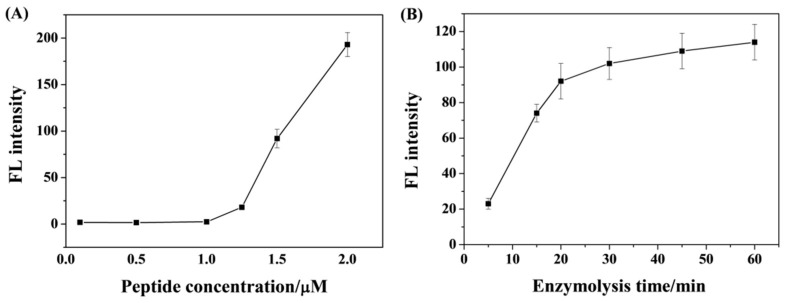
Dependence of fluorescence intensity on peptide concentration (**A**) and enzymolysis time (**B**).

To obtain the best detection performances, the experimental conditions, including peptide concentration and enzymolysis time, were explored. Low background signal can favor the sensitivity of an analytical method. We first investigated the dependence of the fluorescence signal on the peptide concentration. It was found that the fluorescence signal was close to the background when the peptide concentration was below 1 μM (Figure 2A). Beyond this value, the fluorescence intensity began to raise with the increase in peptide concentration. The signal began to increase, implying that the surface of Ni-NTA-MNP was saturated by the peptide probe. In this work, a compromising concentration of peptide was used in light of the real level of Cas3 in samples and the low signal-to-noise ratio. We also found that the fluorescence intensity increased and then reached a platform with the increase of enzymolysis time (Figure 2B). To achieve sensitive and rapid assays, 30 min was chosen as the reaction time for the cleavage event.

### 2.4. Analytical Performances

**Figure 3:**
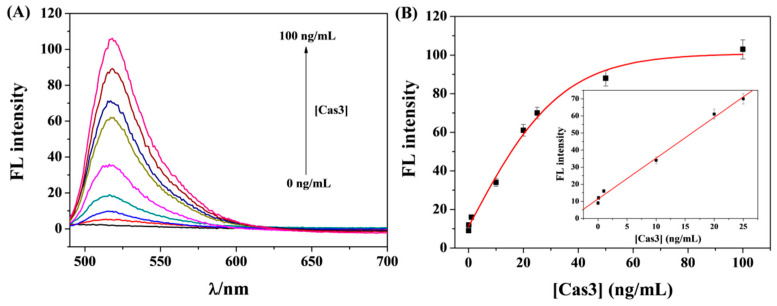
(**A**) Fluorescence spectra for the assays of various concentrations of Cas3 (from bottom to top: 0, 0.01, 0.1, 1, 10, 20, 25, 50 and 100 ng/mL). (**B**) Calibration curve between fluorescence intensity and Cas concentration.

Under optimal conditions, we investigated the analytical performances of this method by determining different concentrations of Cas3. As shown in Figure 3A, the fluorescence signal increased with the increase of Cas3 concentration. A good linear relationship between the fluorescence intensity and Cas3 concentration was found in the range of 0.01–25 ng/mL (Figure 3B). The linear equation is expressed as F = 11.4 + 2.4[Cas3] (ng/mL). The limit of detection (LOD) was found to be 4 pg/mL, which is lower than that of other fluorescence assays with nanomaterials as the quenchers and carriers to load the peptide substrates (Table 1). The sensitivity is also comparable to that achieved by the heterogeneous methods through immobilization of peptides on a solid surface for enzymatic cleavage [49,50]. The high sensitivity should be attributed to the low background signal and high cleavage efficiency of this proposal.

### 2.5. Selectivity

**Figure 4:**
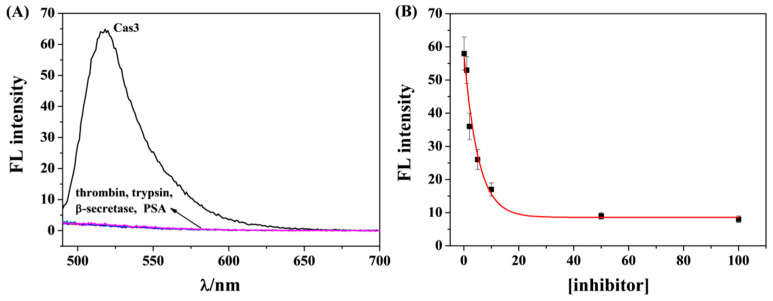
(**A**) Selectivity of this method toward 20 ng/mL Cas, thrombin, trypsin, β-secretase and PSA. (**B**) Dependence of fluorescence intensity on inhibitor concentration.

To explore the selectivity, a series of proteases were tested, including Cas3, thrombin, trypsin, β-secretase and PSA. As a result, only Cas3 caused the enhancement of fluorescence intensity (Figure 4A). Other proteases did not induce a significant signal change. The good selectivity of the proposal can be attributed to the high specificity of the peptide toward Cas3 and the unique interaction between His-tag and Ni-NTA. We also investigated the influence of other possible components in biological samples such as serum on the detection of Cas3. It was found that there is no significant difference in the signals for the assays of Cas3 in buffer and diluted serum. This result is acceptable since the commercialized Ni-NTA-MNPs were highly specific to His-tag peptides or proteins and only a few natural species with His-tag exist in serum at most.

To further confirm that the method is highly specific to active Cas3, the inhibition efficiency of a well-known inhibitor (DEVD-FMK) was evaluated. With the increase ininhibitor concentration, the fluorescence intensity decreased gradually and finally began to level off (Figure 4B). This demonstrated that the inhibitor prevented the cleavage of peptides by depressing the enzymatic activity of Cas3. Based on the relationship between fluorescence intensity and inhibitor concentration, the half-maximum inhibition value (IC50) was found to be 4.6 nM for 20 ng/mL Cas3. The inhibition efficiency was in agreement with that evaluated by other methods [59,60,61,62], suggesting that the method has a promising application for rapid and high-throughput screening of protease inhibitors.

### 2.6. Evaluation of Cell Apoptosis

**Figure 5:**
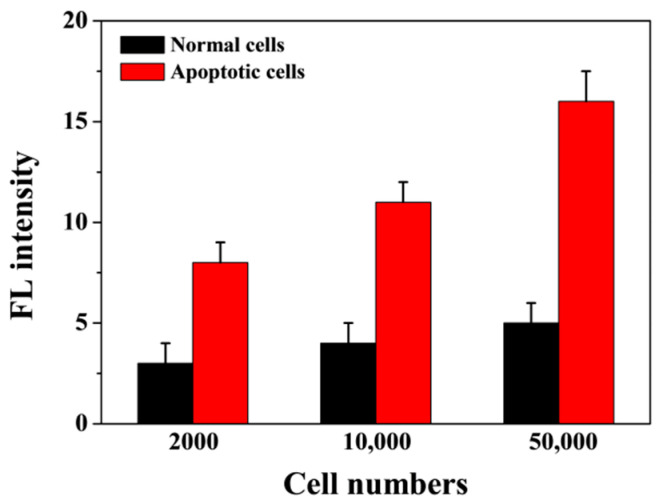
Results for the detection of Cas3 in the normal and apoptotic cells.

To indicate the applicability of this proposal, apoptosis was evaluated by monitoring the activity of Cas3 in the cell lysates. MCF-7 cells were induced to apoptosis with a classical inducer staurosporine. As shown in Figure 5, the fluorescence intensity was significantly intensified with an increasing number of cells induced with staurosporine. However, a slight change was observed for the assays of cells without treatment with the inducer. This result suggests that the level or activity of Cas3 in the apoptotic cells is higher than that in the living cells, which is consistent with that of previous reports [59,60]. Therefore, the proposed method could be used to determine Cas3 in biological samples and evaluate cell apoptosis, showing great potential for developing therapeutic drugs toward apoptosis-related diseases.

## 3. Experimental

### 3.1. Chemicals and Reagents

Cas3, β-secretase and thrombin were ordered from Sigma-Aldrich (Shanghai, China). Trypsin was provided by Sangon Biotech. Co., Ltd. (Shanghai, China). Prostate-specific antigen (PSA) was obtained from Linc-Bio Science Co., Ltd. (Shanghai, China). Peptides were synthesized with a solid phase synthesis method and purified using ChinaPeptides Co., Ltd. (Shanghai, China). NTA-Ni-MNPs with an average size of 400 nm were purchased from PuriMag Biotech. (Xiamen, China). Other reagents were of analytical grade and used without further purification. All the aqueous solutions were freshly prepared with ultrapure water treated with a Millipore system.

### 3.2. Procedures for Cas3 Detection

A total of 100 μL of peptide substrate with the sequence of FITC-GDEVDGHHHHHH (denoted as FITC-GDEVDGH_6_) was incubated with 100 μL of Cas3 in phosphate buffer (10 mM, pH 7.4) at room temperature for a given time. Following this, 10 μL of 12.5 mg/mL Ni-NTA-MNP suspension was added to the reaction solution and shaken for 5 min. Under the action of magnetic force, the supernatant solution was removed for fluorescence measurement on a fluorescence spectrometer with an excitation wavelength of 470 nm.

To evaluate the inhibition efficiency, the inhibitor was incubated with Cas3 for 10 min. Then, the mixture of Cas3 and inhibitor was incubated with the peptide substrate following the above procedure. The inhibition efficiency was calculated according to the change in fluorescence intensity.

### 3.3. Apoptosis Analysis

The extraction of cell lysates from living and apoptotic MCF-7 cells followed the procedure in our previous reports [59,60]. The collected lysates were diluted at different times with phosphate buffer and then analyzed with the steps as those for the assays of Cas3 standard samples.

## 4. Conclusions

In summary, a sensitive and simple homogeneous fluorescence method was proposed for the determination of proteases with Cas3 as the model analyte. The influence of steric hindrance on the enzymatic efficiency was also evaluated. Our proposal does not require the pre-immobilization of the peptide substrate, simplifying the operation steps and improving the sensitivity. The LOD of the method is lower than that of other fluorescence assays via immobilization of substrates on solid surfaces and comparable to that of heterogeneous strategies with signal amplifications of enzymes or nanomaterials. The method was successfully used to evaluate inhibition efficiency and monitor cell apoptosis. Other protease biosensors may be readily designed with this proposal by matching the sequence-specific peptide substrates and signal reporters.

## Figures and Tables

**Table 1:** Analytical performances of nanomaterial-based fluorescence assays of Cas3.

Materials/Reporters	Linear Range	LOD	Ref.
AuNPs/dye	0–1 unit/mL	0.0079 unit/mL	[51]
AuNPs/dye	0–300 ng/mL	0.073 ng/mL	[52]
AuNPs/dye	3.2–100 pg/mL	1.3 pg/mL	[53]
AuNPs/SiNPs	0.05–1.0 U/mL	0.05 U/mL	[54]
GO/dye	7.25–362 ng/mL	7.25 ng/mL	[55]
GO/dye	2–360 ng/mL	0.33 ng/mL	[56]
qPNPs/dye	2–40 nM	0.09 nM	[57]
MCN/dye	0.01–1 ng/mL	0.4 pg/mL	[58]
MB/dye	0.01–25 ng/mL	4 pg/mL	This work

Abbreviations: AuNPs, gold nanoparticles; SiNPs, [Ru(bpy)_3_]^2+^–encapsulated silica nanoparticles; GO, graphene oxide; qPNPs, polymeric nanoquenchers; MCN, mesoporous carbon nanospheres.

## Data Availability

Not applicable.
